# Male or Female? - Influence of Gender Role and Sexual Attraction on Sex Categorization of Faces

**DOI:** 10.3389/fpsyg.2021.718004

**Published:** 2021-09-21

**Authors:** Teresa Luther, Carolin A. Lewis, Melina Grahlow, Philippa Hüpen, Ute Habel, Celia Foster, Isabelle Bülthoff, Birgit Derntl

**Affiliations:** ^1^Department of Psychiatry and Psychotherapy, Tübingen Center for Mental Health (TüCMH), University of Tübingen, Tübingen, Germany; ^2^Emotion Neuroimaging Lab, Max Planck Institute for Human Cognitive and Brain Sciences, Leipzig, Germany; ^3^International Max Planck Research School on Neuroscience of Communication: Function, Structure, and Plasticity, Leipzig, Germany; ^4^Graduate Training Centre of Neuroscience, University of Tübingen, Tübingen, Germany; ^5^Department of Psychiatry, Psychotherapy and Psychosomatics, RWTH Aachen University, Aachen, Germany; ^6^Institute of Neuroscience and Medicine, JARA-Institute Brain Structure Function Relationship (INM 10), Research Center Jülich, Jülich, Germany; ^7^Biopsychology and Cognitive Neuroscience, Faculty of Psychology and Sports Science, Bielefeld University, Bielefeld, Germany; ^8^Department of Human Perception, Cognition and Action, Max Planck Institute for Biological Cybernetics, Tübingen, Germany; ^9^LEAD Graduate School & Research Network, University of Tübingen, Tübingen, Germany; ^10^International Max Planck Research School for Cognitive and Systems Neuroscience, University of Tübingen, Tübingen, Germany; ^11^Tübingen Neuro Campus, University of Tübingen, Tübingen, Germany

**Keywords:** face perception, sex categorization, gender role, gender, sexual orientation

## Abstract

The categorization of dominant facial features, such as sex, is a highly relevant function for social interaction. It has been found that attributes of the perceiver, such as their biological sex, influence the perception of sexually dimorphic facial features with women showing higher recognition performance for female faces than men. However, evidence on how aspects closely related to biological sex influence face sex categorization are scarce. Using a previously validated set of sex-morphed facial images (morphed from male to female and vice versa), we aimed to investigate the influence of the participant’s gender role identification and sexual orientation on face sex categorization, besides their biological sex. Image ratings, questionnaire data on gender role identification and sexual orientation were collected from 67 adults (34 females). Contrary to previous literature, biological sex *per se* was not significantly associated with image ratings. However, an influence of participant sexual attraction and gender role identity became apparent: participants identifying with male gender attributes and showing attraction toward females perceived masculinized female faces as more male and femininized male faces as more female when compared to participants identifying with female gender attributes and attraction toward males. Considering that we found these effects in a predominantly cisgender and heterosexual sample, investigation of face sex perception in individuals identifying with a gender different from their assigned sex (i.e., transgender people) might provide further insights into how assigned sex and gender identity are related.

## Introduction

As social beings, humans communicate with each other on an almost daily basis and research has found that even before communication is initiated, we are able to derive crucial information about our fellow human beings solely based on their faces – regarding characteristics such as age (Bruce and Young, 1986; Wiese et al., 2008; Rhodes, 2009; Carbon et al., 2013), identity (Haxby et al., 2000; Schyns et al., 2002; Calder and Young, 2005), sexual orientation (Rule et al., 2008, 2009; Tskhay et al., 2013) and biological sex (Bruce et al., 1993), and also regarding emotions (Ekman et al., 1987; Prkachin, 2003; Bombari et al., 2013), personality traits (Winston et al., 2002; Willis and Todorov, 2006) and attractiveness (Perrett et al., 1998; Little et al., 2011). Extraction of this socially relevant information might guide our behavior in interpersonal situations and determine the way we approach our counterparts. However, to be able to perform categorization tasks from faces, facial information first needs to be encoded. For sex categorization specifically, the encoding process, and thus accuracy in performing the task, seems to be strongly influenced by featural cues of the face itself, but is also influenced by several attributes related to the individual perceiving the face (Smith et al., 2007; Hillairet de Boisferon et al., 2019).

Literature concurrently indicates that face processing relies on diagnostic information from configurations between individual facial features (e.g., the distance between mouth and nose) as well as on information gained from facial features themselves, such as the mouth and nose (McKelvie, 1976; Rakover, 2012). With regards to sex categorization, various studies provide evidence that featural information from the eye region is particularly relevant for performance in sex categorization tasks (Schyns et al., 2002; Armann and Bülthoff, 2009; Dupuis-Roy et al., 2009). Using Bubbles (Gosselin and Schyns, 2001) – a frequently applied technique that involves partial masking of face stimuli to isolate the facial information that is used to resolve sex categorization tasks – Schyns et al. (2002) demonstrated in a sample of students that information from certain facial features is selectively used depending on the categorization task. They found that information contained in the eye region, especially in the left eye, is particularly important for performing sex categorization. For identity judgements, on the other hand, participants relied on information from both the eye and the mouth region. Similar results were obtained using morphed face stimuli in a study by Armann and Bülthoff (2009), wherein participants were presented with two face images simultaneously, varying either along an identity or a sex morphing continuum, and had to perform sex and identity judgements. Upon investigation with eye-tracking, in both discrimination tasks, fixations were mainly directed toward the eyes – an effect that was even stronger in the sex discrimination task. Although accuracy rates found by Dupuis-Roy et al. (2009) confirm the eye-eyebrow area as the most important region for sex categorization, they also suggest that we only rely on luminance cues from the eyes when color information from the mouth region is not available. Additional evidence that luminance contains diagnostic information for sex classification is provided by Russell (2009), who found that female faces exhibit greater luminance contrast in the eye and lip region as well as in the surrounding skin compared to male faces. In addition, an androgynous face was perceived as more feminine when the luminance contrast was increased. Furthermore, luminance contrast in female faces was increased even more through the application of makeup. Apart from featural information, categorization with regard to sex also decisively depends on diagnostic information from configurations and structure within a face. For example, Roberts and Bruce (1988) showed that categorization was impaired the most, when the nose was obscured in whole faces, however, when presented in isolation, least information was gained from the nose. The authors conclude from these findings that reliable information about the sex of a face is not gained from the nose alone but rather from configurations that are lost when the nose region is obscured. Taken together, diagnostic information regarding the sex of a face is conveyed in the local features as well as in the configurations between those single features and in structural cues (see also Bruce et al., 1993).

Besides the influence of featural and configurational information, sex categorization from faces has been related to certain attributes on the part of the perceiver, such as their biological sex. A common finding is that girls and women show an *own-sex bias*, i.e., higher accuracy in recognizing female faces compared to males faces, while evidence for an equivalent *own-sex bias* displayed by male perceivers for male faces is rather scarce (Cross et al., 1971; Lewin and Herlitz, 2002; Rehnman and Herlitz, 2006, 2007). A frequently discussed explanation for why this effect usually only occurs in girls and women is a greater exposure of both, infant girls and infant boys, to female caregivers in early childhood leading to a superior familiarity with female faces (Herlitz and Lovén, 2013). This female face advantage is further strengthened in girls by the fact that they, in general, attend to faces to a greater extent than boys (Connellan et al., 2000). In combination with gender-specific imitations, this results in a more prominent orientation toward other females as interaction partners (Lewin and Herlitz, 2002; Herlitz and Lovén, 2013) and thus to even more experience with female faces compared to male faces. Another effect, which is frequently found in face perception literature on sex categorization, is the so-called *male bias.* This effect refers to the misclassification of female faces as male faces in sex categorization tasks. It has been observed in experimental setups where adult participants categorize sex-unambiguous faces of neonates, children or adults, which are displayed with eyes facing forward (Kaminski et al., 2011; Daniel and Bentin, 2012; Hillairet de Boisferon et al., 2019) as well as where adults categorize sex-ambiguous (Armann and Bülthoff, 2012) or profile-view silhouette faces (Davidenko, 2007). Researchers usually explain this finding by the absence of diagnostic external sex cues, especially hair, which might be perceived as an indication of baldness being more associated with maleness and thus resulting in the male response bias (Davidenko, 2007). However, given the fact that there are some general sex differences in how face stimuli are perceived and processed (Heisz et al., 2013; Proverbio, 2017), and the suggestion of male faces requiring less information to be correctly recognized (Wild et al., 2000; Cellerino et al., 2004), further research is needed to explore potential reasons for the female *own-sex bias* and the male response bias more extensively.

As of yet, little is known about the potential influence of perceiver attributes that are closely related to biological sex, such as gender, sexual orientation, and gender roles. Gender, defined as an individuals’ identification as male or female (VandenBos, 2015), has along with perceivers’ age and ethnicity been found to not significantly influence face gender categorization (Simpkins, 2011). One study assessed the relationship of sexual orientation primarily in the context of performing attractiveness judgements and showed that sexual orientation indeed affected attractiveness judgements – while homosexual men judged masculine faces more attractive compared to heterosexual men, no significant group differences were found in homosexual and heterosexual female participants (Hou et al., 2019). Another study also assessed the influence of sexual orientation on voice perception and showed a higher categorization accuracy in heterosexual male and female individuals for voices of the opposite sex, whereas homosexual individuals performed categorization of same-sex voices with higher accuracy (Smith et al., 2019). Notably, given the paucity of studies on the relationship of face sex categorization and perceiver attributes closely related to their biological sex, the aim of the present study was to investigate how biological sex, gender, sexual orientation, and gender role influence face sex categorization performed on sex-ambiguous face stimuli. Regarding the influence of biological sex, we hypothesized that female participants would recognize original female faces and male faces morphed to female with higher accuracy compared to original male faces and female faces morphed to male, thereby replicating the *own-sex bias*. Furthermore, we expected to replicate the previously reported *male bias* defined as a tendency to misclassify female faces as male. Our hypotheses regarding gender, sexual orientation and gender roles were nondirectional as - to our knowledge – this is the first study assessing how gender, gender roles, and sexual orientation of the perceiver might influence sex categorization performed on sex-ambiguous face stimuli.

## Materials and Methods

### Sample Description

A total of 86 individuals (35 female, 33 male, and 18 not indicating their sex) participated in the study. Data of 19 participants was excluded from analysis as they either did not fully provide sociodemographic information and questionnaire data (*n* = 1), or they rated less than the minimum of 150 images specified prior to the experiment (*n* = 18). All remaining individuals formed the final sample consisting of 67 participants aged 18–63 (*M* = 32.49 years, SD = 12.46 years), 34 of which were female and 33 were male. Of the 67 participants, 55 provided ratings for all 300 images whereas 12 provided ratings for at least 255 images (85% of images). Female and male participants did not significantly differ in terms of age [female: *M* = 29.97 years, SD = 10.54 years; male: *M* = 35.09 years, SD = 13.53 years; and *t*_(65)_ = −1.70, *p* = 0.093]. All participants identified their gender as either explicitly “male” or “female.” One participant, reporting to be biologically male, indicated her gender as female. All participants had normal vision or used visual aids during the experiment, and they provided written informed consent prior to participation. They took part voluntarily and no reimbursement was offered for participation. All participants were asked to take part individually on their computers or laptops. The study protocol was approved by the ethical review board of the Max Planck Society (2016_02). Detailed information on sample characteristics is provided in Table 1.

**TABLE 1:** Sample description.

	*n*	*M* (SD)	Range
Age, years	67	32.49 (12.46)	18 – 63
Sex (female/male/intersexual)	34/33/0		
Gender (female/male/other)	35/32/0		
Ethnical identity (European/South Asian/Central Asian)	65/1/1		
Native language (German/Dari/Nepali/Turkish/Croatian/Russian)	64/1/1/1/1/1		
Relationship status (single/in relationship/married/divorced/widowed)	18/32/15/2/0		
Education level (higher education/high school/vocational training/middle school/craftsman’s diploma/still in school/doctorate	27/24/11/2/1/1/1		

### Procedure

The experiment was conducted online using the software SoSci Survey (Leiner, 2020) and the questionnaire was made available in German only via www.soscisurvey.de. Participants were provided with the link to the experiment by email and after written information about the study, they provided their written informed consent. Subsequently, each participant provided sociodemographic information regarding sex, gender, and gender roles before the actual experiment began. Each participant underwent 300 trials in each of which one face stimulus, randomly selected from the total stimulus pool of 300 faces, and a Visual Analog Scale (VAS) were presented simultaneously in the center of the display. For each of the presented images, participants indicated how male or female they perceived the image by moving a slider along the VAS with scale endpoints labeled *male* and *female*. Stimuli were presented without replacement, so each participant rated each of the 300 faces exactly once. Even though participants were instructed to respond spontaneously, the experiment was self-paced and no reaction times were recorded.

#### Facial Stimuli

**FIGURE 1:**
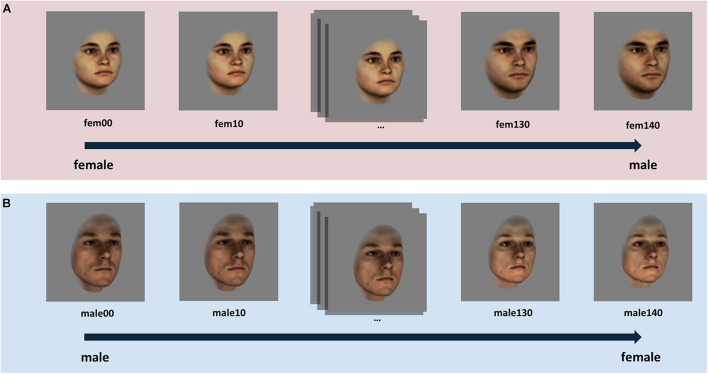
Illustration of the morphing procedure for **(A)** original female face identities and **(B)** for original male face identities, exemplified by means of one female and one male identity. The image captioned “fem00” represents a “superfemale” image which was created by feminizing the original face scan (fem40) of the corresponding female identity. Both the original female face (fem40) and the original male face (male00) were morphed in 10% – intervals along the sex morph continua, created based on a sex vector, to the opposite-sex version endpoint face.

To investigate how female and male participants perceive sex-ambiguous faces, we used a previously validated set of sex-morphed facial images (morphed from male to female and vice versa) which has been created based on 3-dimensional laser scans of real heads from the database of the Max Planck Institute for Biological Cybernetics in Tübingen (Blanz and Vetter, 1999; Armann and Bülthoff, 2012). The stimulus set consisted of 10 original female and 10 original male face identities, morphed to the sex-opposite endpoint in 14 morphing steps. In addition, the images of original female faces were morphed to same sex superfemale because it was evident in a previous rating experiment that the original female faces were not equally perceived as female as the original male faces were perceived as male (see also Armann and Bülthoff, 2012). In order to obtain similar sex ratings for male and female faces (both for the original images and for the opposite-sex versions) super-feminized faces were created only for the original female faces. In total, 300 faces were presented with the hair cropped at the hairline and free of makeup, glasses and facial hair. Faces were presented turned to the right by 20°, in a 24-bit color format on a gray background. Each image was 330 pixels in height and 330 pixels in width. See Figure 1 for an illustration of the face stimuli used.

#### Sex Categorization of Face Stimuli

We used VAS to assess the extent to which each of the presented face stimuli was perceived as male or female. In each trial, a VAS with the left extrema labeled *male* and the right extrema labeled *female*, was displayed underneath each centrally presented face image and participants were asked to indicate the degree of perceived male- or femaleness by moving an initially centrally located slider with the cursor. For analyses, the response of the participants on the sliding scale was translated into a rating ranging from 0 (*male*) to 100 (*female*). Two cut-off values were set *a priori*: ratings from 0 to 20 corresponding to “unambiguously male” and ratings from 80 to 100 corresponding to “unambiguously female.” A rating of 50 was defined as “sex-ambiguous.” We decided to set the cut-offs more extreme compared to the ones set in a previous study (unambiguous maleness and femaleness at values 28.57 and 71.43, respectively; Armann and Bülthoff, 2012) in order to allow for a greater differentiation of the ratings.

#### Questionnaires

Sociodemographic information was obtained from the participants using a self-compiled questionnaire. In addition, subjects completed two self-report inventories: The Gender-Related Attributes Survey (GERAS; Gruber et al., 2019) and the Gender Inclusive Scale (Galupo et al., 2017, 2018).

##### Gender-Related Attributes Survey

The GERAS is a self-report measure that assesses gender role identity based on positive and negative attributes which are typically associated with the female or male gender in middle European cultures (Gruber et al., 2019; Pletzer et al., 2019). The inventory consists of 50 items representing the following three subscales: *Personality*, comprised of 20 items, *Cognition*, comprised of 14 items, and *Interests* & *Activities*, comprised of 16 items. In the subscale *Personality*, participants are asked to indicate how often certain traits apply to them on a 7-point Likert scale ranging from 1 (*never*) to 7 (*always*). In the subscale *Cognition*, they are asked to rate how well they are able to solve certain problems, and in the *Interests* & *Activities* subscale, subjects are asked to indicate how much they like certain activities. Responses to the latter two scales are given on a 7-point Likert scale ranging from 1 (*not at all*) to 7 (*very*). For each of the three subscales, half of the items describe feminine attributes, while the other half describes male attributes. Masculinity and femininity scores can either be calculated for each of the three scales individually by averaging the ratings for masculine and feminine items or for the inventory as a whole by averaging the individual masculinity and femininity scores of the three subscales. In this study, masculinity and femininity scores were analyzed both on scale level as well as overall. Due to technical issues, subscale *Cognition*, originally comprising 14 items, in this study comprised 13 items with one item coding for masculinity missing. Subscale *Interests* & *Activities* in this study comprised 13 out of originally 16 items, missing two items coding for masculinity and one item coding for femininity. The reduced number of items was accounted for in the calculation of scores.

##### Gender Inclusive Scale

The Gender Inclusive Scale is a self-report inventory assessing sexual orientation by taking both sex and gender aspects into account (Galupo et al., 2017, 2018). The scale consists of six items, two of which describe elements based on the dimension sex (attraction to females and males), whereas the other four are related to dimensions of gender (attraction toward masculine, feminine, androgynous, and gender nonconforming individuals). All items are rated on a 7-point Likert scale ranging from 1 (*not at all*) to 7 (*very*). Each item is analyzed individually. No singular overall score is calculated for this inventory.

### Data Analysis

Analyses were conducted using R version 3.5.1 (R Core Team, 2018). Visual inspection suggested that ratings and questionnaire data were normally distributed, therefore, all analyses were conducted using parametric statistical methods with two-tailed significance at *p* < 0.05. Mean differences in femininity and masculinity traits and in sexual orientation of female and male participants were analyzed using *t-*tests for unpaired samples. To compare the proportion of ratings in the margin areas (below 20 and above 80), we used a Chi-Square Goodness of Fit Test. To investigate the potential impact of various attributes of the perceiver, a linear mixed-effects model approach provided by R package lme4 version 1.1.23 (Bates et al., 2015) was used. Firstly, to check whether model assumptions were met, residual distribution was inspected. As this indicated normally distributed residuals, we proceeded with the mixed-model approach. We set up linear-mixed effects models with rating of female- and maleness of the images as the outcome variable, fixed effects of sex of image and morphing level and their interaction (basic model). The responses on the GERAS and the Gender Inclusive Scale were dichotomized. Participants reporting higher overall identification with feminine/masculine attributes were considered as “feminine”/“masculine.” Given the similar distribution of responses to items “attraction to females” and “attraction to feminine individuals” and to items “attraction to males” and “attraction to masculine individuals,” responses to these items were collapsed into the category “attraction to females” and “attraction to females,” respectively. The term *sexual attraction* instead of *sexual orientation* will therefore be used in the following when referring to the results of the Gender Inclusive Scale. To account for the repeated measurement and random variability across participants and stimuli, random effects in participants and male and female images were incorporated (random intercepts). The variable gender was not analyzed individually as only one participant reported to identify with a gender different from their biological sex. We conducted a sensitivity power analysis using G^∗^Power version 3.1.9.7 (Faul et al., 2007) to calculate the critical population effect size to find interaction effects accepting a type II error probability of 20%. Our sample (*N* = 67) was sufficiently powered to detect a medium effect (*f*^2^ = 0.255).

## Results

### Gender Role Identity and Sexual Attraction

We found significant differences between male and female participants with regard to gender role identity. On average, female participants indicated significantly higher overall femininity scores than male participants [females: *M* = 5.05, SD = 0.58; males: *M* = 4.24, SD = 0.54; and *t*_(65)_ = 5.90, *p* < 0.001] and male participants obtained significantly higher overall masculinity scores than female participants [males: *M* = 4.57, SD = 0.66; females: *M* = 3.99, SD = 0.68; and *t*_(65)_ = −3.55, *p* < 0.001]. Furthermore, as can be seen in Table 2, female participants obtained significantly higher femininity scores on subscales *Personality* (*p* < 0.001) and *Interests* & *Activities* (*p* < 0.001) compared to male participants, whereas male participants obtained significantly higher masculinity scores on all three subscales compared to females (all ps < 0.05).

**TABLE 2:** Mean femininity and masculinity scores (and standard deviations) for female and male participants for the Gender-Related Attributes Survey (GERAS) subscales.

Femininity score				

Subscale	Females	Males	*t* (*df*)	*p*
Personality	5.44 (0.58)	4.78 (0.66)	4.25 (65)	<0.001*
Cognition	5.03 (1.01)	4.82 (0.83)	0.92 (65)	0.362
Interests & activities	4.66 (1.08)	3.10 (0.77)	6.71 (65)	<0.001*

**Masculinity score**				

**Subscale**	**Females**	**Males**	***t* (*df*)**	***p***

Personality	3.99 (0.69)	4.46 (0.75)	−2.67 (65)	0.010*
Cognition	4.70 (0.92)	5.27 (0.93)	−2.49 (65)	0.015*
Interests & activities	3.28 (1.06)	3.97 (1.16)	−2.51 (65)	0.015*

*p < 0.05.

Results from the Gender Inclusive Scale suggest that the majority of participants were heterosexual. Male participants indicated to be more attracted toward women and feminine individuals than female participants. Conversely, female participants indicated to be more attracted toward men and masculine individuals compared to male participants. Female participants also indicated higher attraction toward androgynous individuals and toward gender nonconforming individuals compared to male participants All mentioned differences between female and male participants proved to be statistically significant (all *p* ≤ 0.02). For statistical details see Table 3.

**TABLE 3:** Mean differences (and standard deviations) in female and male participants regarding items of the Gender Inclusive Scale.

Item	Females	Males	*t* (*df*)	*p*
I am attracted to women	2.59 (1.76)	6.64 (0.93)	−11.72 (65)	<0.001*
I am attracted to men	6.09 (1.64)	1.45 (1.18)	13.26 (65)	<0.001*
I am attracted to masculine individuals	6.00 (1.26)	1.58 (1.12)	15.21 (65)	<0.001*
I am attracted to feminine individuals	2.68 (1.49)	5.24 (2.12)	−5.74 (65)	<0.001*
I am attracted to androgynous individuals	2.74 (1.81)	1.79 (1.24)	2.49 (65)	0.016*
I am attracted to gender nonconforming individuals	2.18 (1.49)	1.48 (0.83)	2.34 (65)	0.022*

*p < 0.05.

### Image Ratings

Overall, across all identities and participants, images received a mean rating of 39.07, indicating a general bias toward the male end of the rating continuum. Thirty-three percent of images received a rating ranging into the female section (i.e., a rating above 50 on the rating scale) and 67% of images received a rating ranging into the male section (i.e., a rating below 50 on the rating scale). Regarding the margins, a significantly greater proportion of images received ratings from 0 to 20 inclusively than ratings from 80 to 100 inclusively, *χ*^2^(1) = 1677.70, *p* < 0.001. Original male images were rated more male on average (*M* = 17.30, SD = 17.77) than original female faces were rated female (*M* = 57.38, SD = 29.46), and even the mean ratings for the superfemale images did not reach the “unambiguously female” section (*M* = 71.86, SD = 25.48). So, the effect that it is generally harder to classify original female faces as female then it is to classify original male faces correctly as male was replicated.

### Influencing Factors on Ratings

#### Image Characteristics

**FIGURE 2:**
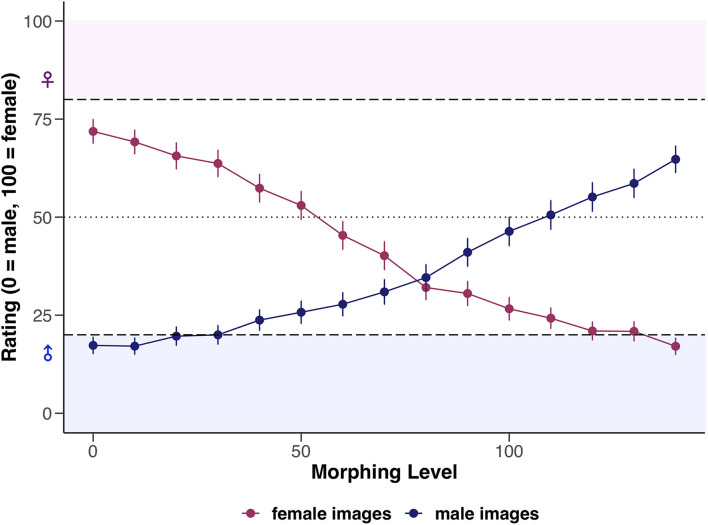
Mean ratings of female and male images depending on morphing level (0–140), visualized separately for female and male participants. Error bars represent standard error (SE). Dashed horizontal lines represent the *a priori* defined cut-off values (ratings between 0 and 20 = unambiguously male, ratings between 80 and 100 = unambiguously female, and ratings of 50 = sex-ambiguous).

The basic model (LMM0, see Table 4) revealed that original male images were generally rated significantly lower by 61.77 units on the rating scale than female faces which have been feminized (i.e., superfemale images). Furthermore, rating of superfemale images significantly decreased by 4.30 units (i.e., the images were rated more male) when morphing increased by one 10%-interval. With each 10%-increase in morphing, rating of original male images significantly increased by 3.52 units. Statistical details of the basic model are reported in Supplementary Material. For an illustration of the interaction between the factors *sex of image* and *morphing level*, see Figure 2.

**TABLE 4:** Relationship between perceiver attributes and rating.

Model	Formula	Tested parameter	χ^2^	*df*	*p*	AIC	BIC
LMM0	Rating_ij_ = β_0_ × β_1_ image_i_ × β_2_ morph_j_ + υ_0id_ + υ_id:image_ + υ_id:morph_ + υ_id:ident_ + ε_ij_					181743	181814
LMM1	Rating_ijk_ = β_0_ × β_1_ image_i_ × β_2_ morph_j_ × β_3_ sex_k_ + υ_0id_ + υ_id:image_ + υ_id:morph_ + υ_id:ident_ + ε_ijk_	β_3_/sex	2.64	4	0.621	181748	181851
LMM2	Rating_ijk_ = β_0_ × β_1_ image_i_ × β_2_ morph_j_ × β_3_ GERAS_k_ + υ_0id_ + υ_id:image_ + υ_id:morph_ + υ_id:ident_ + ε_ijk_	β_3_/GERAS	29.57	4	<0.001*	181721	181824
LMM3	Rating_ijkl_ = β_0_ × β_1_ image_i_ × β_2_ morph_j_ × β_3_ GERAS_k_ × β_4_ GIS_l_ + υ_0id_ + υ_id:image_ + υ_id:morph_ + υ_id:ident_ + ε_ijkl_	β_4_/GIS	57.14	8	<0.001*	181680	181846

*β_0_, mean rating for superfemale images in female participants; β_1_, estimate for the effect of sex of image (original male or female); β_2_, estimate for the effect of morphing (0–140); β_3_, estimate for the effect of sex (male or female) or gender (masculine or feminine) of participants; β_4_, estimate for the effect of sexual attraction (attraction toward males or females); υ_0id_, random effect of participants on intercept of rating; υ_*id:image*_, random effect of participants and image on intercept of rating; υ_*id:morph*_, random effect of participants and morphing on intercept of rating; υ_*id:ident*_, random effect of participants and face identity on intercept of rating; ε_*ij*_, residual variance; image_*i*_, sex of image (original male or female); morph_*j*_, morphing level (0–140); sex_*k*_, sex of participants; GERAS_*k*_, gender of participants (masculine or feminine); GIS_*l*_, sexual attraction of participants (attraction toward males or females); AIC, Akaike information criterion; BIC, Bayesian information criterion; LMM0, basic model; LMM1, model with interaction effect of sex; LMM2, model with interaction effect of gender; and LMM3, model with interaction effect of gender and sexual attraction.*

*p < 0.05.

#### Perceiver Attributes: Sex, Gender Role, and Sexual Attraction

To investigate the potential influence of perceiver attributes on the rating of the images, the following factors were successively added to the fixed interaction as additional predictor variables: Sex, gender (femininity or masculinity indicated on the GERAS), sexual attraction (attraction toward females or males indicated on the Gender Inclusive Scale). First, we tested the basic model against two models with only one additional interaction effect (LMM1 and LMM2, Table 4). Finally, we tested the model with the interaction effect of gender against a model with an additional interaction effect of sexual attraction (LMM3, Table 4). Model comparisons were conducted via likelihood ratio tests.

**FIGURE 3:**
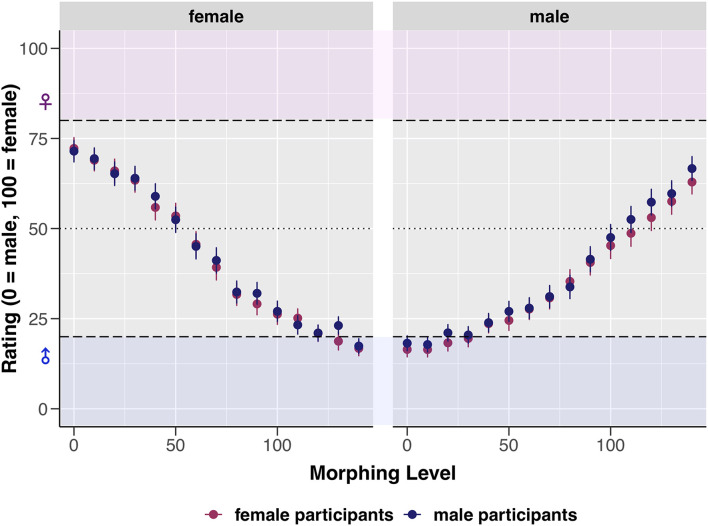
Visualization of the effect of morphing level (0–140) and sex of image (male or female) on the mean ratings. Error bars represent SE. Dashed horizontal lines represent the *a priori* defined cut-off values (ratings between 0 and 20 = unambiguously male, ratings between 80 and 100 = unambiguously female, and ratings of 50 = sex-ambiguous).

Model comparisons revealed no significant effect of the sex of participants on image rating (*p* = 0.621), see Table 4 model LMM1 for statistical details and Figure 3 for a visualization. However, a significant predictive contribution of participants’ gender and sexual attraction to the rating was revealed (see Table 4 models LMM2 and LMM3). With each 10%-increase in morphing, perceivers with predominantly masculine attributes who indicated attraction toward females rated original male images by 21.66 units higher (i.e., more female) and superfemale images by 12.13 units less (i.e., more male). Participants with predominantly feminine attributes who indicated attraction toward males rated original male images by 18.61 units higher (i.e., more female) and superfemale images by 10.60 units less (i.e., more male). The last two items of the Gender Inclusive Scale were not included in these models as additional predictor variables, as only a small proportion of participants indicated attraction by a score of four or above toward androgynous (*n* = 8) and gender-non-conforming individuals (*n* = 1). For statistical details of the models see Supplementary Material.

## Discussion

The aim of the present study was to investigate how certain attributes of the perceiver influence face sex categorization of faces with varying degree of sex-ambiguousness. Participants were presented with original facial stimuli of 10 female and 10 male identities, as well as with facial images created by morphing the level of sex information in these original faces, and were asked to rate the extent to which they perceived each image as female or male. As we were particularly interested in how perceiver attributes affect the ratings of male and female original faces as well as the sex-ambiguous (i.e., morphed) facial images, we assessed participants’ sex, their gender, their gender role identification and their sexual orientation and related them to the ratings. Besides the exploratory investigation of the effects of gender roles and sexual orientation, we hypothesized a sex effect, with females outperforming males for female faces as well as a *male bias*, which has been described in numerous previous studies within the face perception literature as the tendency to misclassify female faces as male faces in sex categorization tasks.

In contrast to our hypothesis and to various previous findings, we did not find a significant effect of participants’ sex on ratings in our sex categorization task. This discrepancy with research findings of an *own-sex bias*, found primarily in female participants, is probably largely due to methodological differences: In previous studies, Cross et al. (1971) and Rehnman and Herlitz (2006) used yearbook portraits and frontal view images, respectively, not controlling for external features such as clothing and hair style, whereas in a study by Lewin and Herlitz (2002) full-face images as well as faces in which hair, ears, jewelry, and face contour had been removed were used. Further, the stimulus material in all of these three studies consisted of sex-unambiguous faces and the studies’ main focus was on recognition memory of male and female faces instead of face categorization.

We did not run separate analyses for how participants’ gender might affect ratings because only one participant, reporting to be biologically male, indicated to identify as female. So, we expect a similar null finding as with participants’ sex. Since according to our knowledge, only one study has dealt with the impact of observers’ gender on image sex categorization so far, using unambiguous male and female face stimuli, future research specifically recruiting transgender and gender nonconforming individuals is needed to further investigate whether and how the interaction between sex and gender is manifested behaviorally in the rating of morphed, sex-ambiguous facial images. As a result of transgender individuals’ lacking identification with their sex and based on brain-imaging studies demonstrating similarities of transgender individuals with individuals belonging to the gender they desire (e.g., Junger et al., 2014), it would be particularly interesting to assess whether a *(own-gender) bias* similar to the often reported *own-sex bias* in cisgender individuals (e.g., Lewin and Herlitz, 2002) can be found in transgender individuals when they are asked to discriminate sex-ambiguous facial images. Such a research approach would not only provide further insight into how assigned sex and gender identity are related, but also allow for gaining more knowledge about the implications associated with an experienced discrepancy between assigned sex and desired gender.

Regarding gender role identification, male participants indicated that they identified significantly more with masculine attributes on all three subscales of the GERAS compared to female participants. In contrast, female participants stated that they identified significantly more with feminine attributes on GERAS subscales *Personality* and *Interests* & *Activities* compared to male participants. Interestingly, female and male participants did not significantly differ with regard to the obtained femininity score on subscale *Cognition*. The almost equally high scores may be explained by a presumably large overlap of some items, coding for feminine cognitions, with academic skills (“to find the right words to express a certain content,” “to phrase a text,” “to find synonyms for a word in order to avoid repetitions,” “to explain foreign words”).

Based on our fitted models, we found that rating of sex-ambiguous face images is significantly influenced by certain attributes related to gender role identification. For both masculine participants attracted to females and feminine participants attracted to males, the rating of superfemale images decreased with morphing, whereas the rating of original male images increased with morphing. This effect was more pronounced in masculine compared to feminine participants.

In line with previous studies and in accordance with our expectations, we found an overall bias to respond with lower ratings (below 50), i.e., to classify faces more often as male than as female: only one third of images (33.4%) received a rating above 50, while two thirds (66.6%) were rated below 50. Furthermore, our results show that original male faces were rated more strongly as male than original female faces as female. Various potential factors account for this *male bias*, mainly originating from the specificity of the stimulus material, which have been discussed in prior studies. While it could be argued that our stimulus material did not adequately represent sexually dimorphic facial features, the unidirectionality of this response bias is most likely attributable to an interplay of numerous reasons and cannot simply be explained by this general assumption. More specifically, it might be that physiognomic features prototypically associated with female faces, such as a less protrusive, more roundly shaped nose and more protruding eyes compared to men (Enlow and Moyers, 1982), were not distinctive enough in the original female faces, and that morphing of original male faces did not work equally well as the morphing of original female faces. Another sexually dimorphic feature which might have been less visible in our stimuli – although the morphing technique used to create the images (Blanz and Vetter, 1999) allows for adjusting facial texture – is facial contrast (Russell, 2009). Naturally, luminance contrast between the eyes, lips and the surrounding skin is greater in female compared to male faces (Russell, 2009), and it has been shown that the application of makeup can further increase facial contrast, thus making the face perceptually look even more feminine (Cox and Glick, 1986; Russell, 2011). Because the face stimuli in our study were free of makeup, facial contrast could probably not be used as a reliable feature to distinguish female from male faces which might at least partly account for the replication of the *male bias*. Furthermore, note that due to cropping at the hairline, several other cues such as hair style, clothing and jewelry were not visible in our face stimuli. However, it might be that those external features are particularly relevant for recognizing faces an individual has no prior experience with, i.e., unfamiliar faces (Ellis et al., 1979) and more specifically for categorizing female faces (e.g., Burton et al., 1993). Taking into consideration that people construct mental representations of faces through experience and that faces appearing more similar to these prototypical faces are perceived as more familiar and attractive (e.g., Langlois and Roggman, 1990; Rhodes et al., 2003; Halberstadt, 2006), it might be that at least some of the female faces used in our study did not resemble the prototypical female face our participants had formed by experience with female faces in their personal surroundings (e.g., their girlfriends or spouses), encounters with women in everyday life or female faces displayed in media. Also given the fact that we did not select female faces for our study on the basis of their femaleness, such assumptions and expectations formed by the participants might have contributed to the misclassification of female faces.

Another potential factor that might have contributed to our results is the frequently reported link between face femininity and perceived attractiveness: Both male and female observers judge female faces with exaggerated female features, as well as feminized male faces, as more attractive (e.g., Rhodes et al., 2000). Therefore, the effect that female faces were more often misclassified as male might have been further enhanced either by a reduced perceived attractiveness of the stimuli (potentially by not, or only to a lesser degree, displaying features associated with femininity) or by the fact that this reduced attractivity was due to a greater cognitive effort associated with the classification of sex-ambiguous faces compared to the original male and (super-)female faces (Owen et al., 2016). It could also be that at least a small proportion of men whose faces were included in the database displayed a beard shadow, which would also have been reflected in the sex vector, i.e., the difference vector between the average male and the average female face, resulting in female faces that are morphed along the sex vector to the male end of the continuum displaying this slightly male feature already at a low morphing level. While such a visibility of male cues would provide an explanation for the remark given by a few participants that some faces seemed to have beard hair, it could certainly not individually account for the *male bias*, as has already been suggested by Bruce et al. (1993).

### Limitations

Because we did not explicitly control for facial expressions in the images, it is possible that some of the faces used in our study did not display a fully neutral facial expression. However, it is an open question whether this would have been due to the face stimuli we used or whether factors such as lighting or individual face shape could have induced the perception of facial expressions. Several studies indicate an overlap of sex cues and emotional face expressions, in particular that recognition of angry male and happy female faces is enhanced, and that classification of female faces expressing anger is impaired (Becker et al., 2007; Bayet et al., 2015). Thus, depending on what emotions were expressed or perceived in some of the faces, this might have influenced our results in the way that perceived anger could have resulted in judging faces more frequently as male.

Another important aspect that was not controlled for in our study due to the setup of the experiment as an online study, was image size and quality. Although all images were uploaded in 24-bit color format with a pixel size of 330×330, the actual (physical) size in which each image was then displayed on participants’ devices strongly depended on the pixel density (resolution) of the respective end device. Especially the perception of fine facial features such as luminance contrast might therefore have been impaired in some participants and thus could have influenced their ratings.

Finally, we have limitations concerning the assessment of sexual orientation. Even though we designed the study in a way that allows sexual diversity to be represented, by implementing the Gender Inclusive Scale as an instrument that incorporates aspects of both sexual attraction based on sex and based on gender expression, our final sample predominantly comprised of heterosexual individuals.

## Conclusion

Although the present study failed to show a significant effect of participants’ sex on classification of sex-ambiguous facial stimuli, it demonstrates that sex classification performed on sex-ambiguous faces is more strongly impacted by attributes which are closely related to biological sex, such as gender role identification (i.e., gender specific traits) and sexual attraction of perceivers. Given the crucial role of sex categorization for social behavior and interaction, the findings of this study highlight the importance for future research to further investigate the association between perceiver attributes and face sex perception, particularly with regard to the gender and gender role of the perceiver. The application of a similar research design in a sample of transgender and gender nonconforming individuals might help to provide more insight into how assigned sex and gender identity are related, thus allowing us to derive potential implications on how to provide support to these individuals.

## Data Availability Statement

The raw data supporting the conclusions of this article will be made available by the authors, without undue reservation.

## Ethics Statement

The studies involving human participants were reviewed and approved by the Ethical Review Board of the Max Planck Society (2016_02). The patients/participants provided their written informed consent to participate in this study.

## Author Contributions

UH and BD devised the study and the main conceptual ideas. IB and CF developed and validated the stimulus material. TL collected the data and prepared the first draft of the manuscript. TL, CL, and MG performed the data analyses. All authors contributed to designing the study, critically revising and editing the content of the manuscript, and approved the final version of the manuscript for submission.

## Conflict of Interest

The authors declare that the research was conducted in the absence of any commercial or financial relationships that could be construed as a potential conflict of interest.

## Publisher’s Note

All claims expressed in this article are solely those of the authors and do not necessarily represent those of their affiliated organizations, or those of the publisher, the editors and the reviewers. Any product that may be evaluated in this article, or claim that may be made by its manufacturer, is not guaranteed or endorsed by the publisher.
