# Supplementary material for: Male or Female? - Influence of Gender Role and Sexual Attraction on Sex Categorization of Faces
Source: Front Psychol. 2021 Sep 21;12:718004. doi: 10.3389/fpsyg.2021.718004 (PMC8490621; doi:10.3389/fpsyg.2021.718004)
Supplement: Supplementary file 1 [file Data_Sheet_1.docx]

Supplementary Material

## Supplementary Tables

**Supplementary Table 1.** Fixed Effects Estimates of the Null Model.

| **Parameter** | **β** | ***t*** | **95% CI** |
| --- | --- | --- | --- |
| intercept | 72.71* (1.30) | 55.77 | [70.13, 75.29] |
| imagemale | -61.77* (0.95) | -64.86 | [-63.65, -59.89] |
| morph | -4.30* (0.06) | -76.40 | [-4.41, -4.19] |
| imagemale:morph | 7.82* (0.07) | 113.43 | [7.69, 7.96] |

Note: Standard errors of the estimates are given in parentheses. CI = confidence interval; Intercept = superfemale images (reference); imagemale = original male images; morph = morphing level (0-140). * The confidence interval indicates significance at the 0.05 level.

**Supplementary Table 2.** Fixed Effects Estimates of the Model with Interaction of GERAS and Gender Inclusive Scale.

| **Model** | **Parameter** | **β** | ***t*** | **95% CI** |
| --- | --- | --- | --- | --- |
| LMM3 | intercept imagemale morph GERASMas  GISMas imagemale:morph imagemale:GERASMas morph:GERASMas imagemale:GISMAS  morph:GISMas  GERASMas:GISMas  imagemale:morph:GERASMas  imagemale:morph:GISMas  imagemale:GERASMas:GISMas  morph:GERASMas:GISMas  imagemale:morph:GERASMas:GISMas | 78.20* (2.95)  -71.43* (2.15)  -4.91* (0.13)  -8.19* (3.72)  -6.28 (3.57)  8.98* (0.16)  14.42* (2.71)  0.96* (0.16)  10.91* (2.60)  0.58* (0.15)  11.59 (6.79)  -1.74* (0.20)  -1.28* (0.19)  -19.47* (4.95)  -0.83* (0.29)  2.19* (0.36) | 26.52 -33.22 -38.37 -2.20 -1.76 57.20  5.32  5.99  4.20  3.76  1.71  -8.79  -6.75  -3.94  -2.82  6.09 | [72.37, 84.04] [-75.68, -67.91]  [-5.16, -4.66] [-15.55, -0.83]  [-13.34, 0.78]  [8.67, 9.29]  [9.06, 19.77]  [0.65, 1.28]  [5.77; 16.04]  [0.28; 0.88]  [-1.85; 25.03]  [-2.13; -1.35]  [-1.65; -0.91]  [-29.24; -9.69]  [-1.41; -0.25]  [1.49; 2.90] |

Note: Standard errors of the estimates are given in parentheses. GERAS = Gender-Related Attributes Survey; CI = Confidence interval; Intercept = superfemale images (reference); imagemale = original male images; morph = morphing level (0-140); GERASMas = masculine gender identity; GISMas = sexual attraction towards males. * The confidence interval indicates significance at the 0.05 level.
